# Introducing the Concept of Small Vulnerable Newborns (SVN) as an Additional Endpoint to Evaluate Birth Outcomes in the Context of RSV Immunization Studies—A Commentary

**DOI:** 10.1111/irv.70244

**Published:** 2026-03-05

**Authors:** Jacob Gerstenberg, Carlotta Helbig, Benjamin T. Schleenvoigt

**Affiliations:** ^1^ Institute of Infectious Diseases and Infection Control Jena University Hospital, Friedrich‐Schiller‐University Jena Germany; ^2^ Department of Internal Medicine DIAKOVERE Friederikenstift Hannover Germany

## Abstract

Infection with respiratory syncytial virus (RSV) represents a substantial burden of disease, especially in children. While one vaccine (RSVpreF) has been recommended for administration in pregnant women, another one (RSVPreF3‐Mat) has been discontinued in a Phase 3 study due to an increased incidence of preterm births in the study group. When comparing the studies related to these two vaccine candidates, the differences in preterm birth rates appear inconsistent. For a more differentiated evaluation of birth outcomes associated with maternal RSV vaccination, we recommend introducing the previously described concept of “small and vulnerable newborns.”

With great confidence, we have followed the development and recent introduction of maternal vaccines against the respiratory syncytial virus (RSV). RSV causes acute lower respiratory infections, with particularly high consequences in infants and young children, and represents a substantial burden of disease [1]. Preventive strategies against RSV include passive immunization of infants either via direct administration of monoclonal antibodies or by active vaccination of the pregnant mothers. For the latter, there have been two different promising vaccine candidates tested, which both target the RSV prefusion F protein. While one of them has been recommended for vaccination in pregnant women (RSVpreF) [2], the Phase 3 study of the other candidate (RSVPreF3‐Mat) had been stopped due to an increased number of preterm births in the intervention arm [3].

In the MATISSE trial (Maternal Immunization Study for Safety and Efficacy), Madhi et al. investigated the effectiveness of RSVpreF against severe RSV‐associated lower respiratory tract illness in infants as well as birth outcomes [4]. A total of 7386 pregnant participants were included in the study, from which 3698 received RSVpreF and 3688 received placebo. Regarding adverse birth outcomes, the authors reported a preterm birth rate of 5.7% in the intervention group compared to 4.7% in the placebo group (RR 1.20 [0.98–1.46]). While this main analysis showed no significant difference in preterm birth, the stratification among regions of origin showed that the same applies in high‐income regions (preterm birth rate ~5%), whereas in low‐ and middle‐income countries (LMIC), the preterm rate was significantly higher in the RSVpreF group (7.0%) compared to the placebo group (4.0%) with a relative risk of 1.73 [1.22–2.47].

In contrast, Dieussaert et al. published the results from the Phase 3 “RSV MAT‐009” trial investigating RSVPreF3‐Mat among 5328 pregnant women and 5233 newborns [3]. Women were randomized in a 2:1 ratio to intervention and placebo. Due to an observed increased risk for preterm birth in an interim analysis, the recruitment and intervention of the trial were stopped in February 2022. In the post hoc analysis, the authors reported a significantly higher rate of preterm birth of 6.8% (237/3494) in the intervention group compared to 4.9% (86/1739) in the placebo group (RR 1.37 [1.08–1.74]; *p* = 0.01). However, the increased incidence of prematurity in the intervention group was limited to a defined period of time (April–December 2021). As in the MATISSE study, the stratification among regions of origin showed that the increased risk for preterm birth was predominantly observed in LMIC (9.8% vs. 6.3%, RR 1.56 [1.17–2.09]), where approximately 50% of the trial population was enrolled. Meanwhile, the observed incidence of preterm birth in the RSVPreF3‐Mat study group was below the known background incidence for prematurity in 22/24 countries (exception of Mexico and Honduras). In summary, the authors stated that “the increased risk of pre‐ term birth in the vaccine group was largely seen in low‐ and middle‐income countries, was limited to a defined period in the trial, and remains unexplained” [3].

When comparing the reported risks for preterm birth in both studies, it appears that both find an increased risk for preterm birth, but the decisive difference only lies in the fact that in RSV MAT‐009, the lower boundary of the 95% confidence interval falls just below 1, while in MATISSE, it is slightly above (RR 1.20, 95% CI, 0.98–1.46 vs. RR 1.37 [1.08–1.74]). This difference seems less pronounced than expected—given that one of the two vaccines has been approved while the other has not. The difference between both vaccine candidates becomes more doubtful when looking at the results of further Phase 2b (RSV MAT‐004) and Phase 3 studies (RSV MAT‐0012) using RSV PreF3‐Mat, where a possible increased risk for prematurity was not observed. RSV MAT‐004 reported a preterm birth rate of 2.9% in the study group and 3% in the placebo group (RR 0.94, 95% CI, 0.18–5.02). RSV MAT‐012 investigated women with high‐risk pregnancies and found a preterm birth rate of 18.2% in the vaccine group and 19.7% in the placebo group (RR 0.92, 95% CI, 0.50–1.69) [5]. However, both trials had a much smaller sample size (*n* = 504 and *n* = 300) and were not powered to detect differences in prematurity.

A recently published meta‐analysis from Saad et al. [6] included safety data from six different randomized controlled studies including 10,212 pregnant individuals vaccinated with RSVpreF and found no increased risk for preterm birth (OR: 1.09 [0.87–1.37]). Two other large post‐licensure studies (*n* = 24,891 and *n* = 14,099, respectively) confirm these findings (IRR: 0.97 [0.89–1.06] and OR: 0.90 [0.80–1.00], respectively) [7, 8].

Nevertheless, adverse birth outcomes should be thoroughly assessed in the context of maternal immunization studies. While prematurity is an important marker for neonatal morbidity and represents a significant risk factor for the future development of the newborn, other concepts should not be left aside in this matter. One important endpoint represents being small‐for‐gestational‐age (SGA), which usually is defined as fetal birth weight below the 10th percentile for gestational age. To measure this, gestational age should ideally be confirmed by ultrasound findings, and SGA should be calculated using sex‐specific standardized growth‐charts (e.g., from the INTERGROWTH‐21st project) [9]. Both studies have included the investigation of SGA into their main analysis and found no significant difference between study groups.

However, both endpoints prematurity and SGA can also be investigated together. For this purpose, we propose to introduce the concept of “small vulnerable newborns” (SVN) into the analysis of adverse birth outcomes in the context of RSV vaccination safety analysis. The concept of SVN has been previously introduced by a collaborative UNICEF‐WHO group and consists of three groups: preterm non‐SGA, term SGA, and preterm SGA [10]. More than half of the 22.4 million neonatal deaths worldwide fall into one of these groups, and preterm‐SGA newborns showed the highest relative neonatal mortality risk [11]. These findings particularly apply in LMIC, where SVN prevalence was estimated to affect one in four newborns (prevalence 23.91%, neonatal mortality: adjusted RR = 17.51 [95% CI, 12.95–23.67]) [12].

To our knowledge, there are two ongoing noninterventional post‐licensure registries by the manufacturer (NCT06521944 and C3671027) and three ongoing and planned RCTs (NCT07279298, NCT06866405, and NCT06955728) investigating RSVpreF in pregnant women. Including the concept of “small vulnerable newborns” into these and future studies analyzing the safety of maternal RSV vaccines would give a more differentiated assessment on the impact of the vaccine on birth outcomes. This would particularly benefit the most at‐risk newborns and those living in LMIC, where prematurity was mostly observed in this context, where the burden of RSV infection is highest, and where the potential benefit of the vaccine may be greatest.

## Author Contributions

**Jacob Gerstenberg:** writing – original draft. **Carlotta Helbig:** writing – original draft. **Benjamin T. Schleenvoigt:** conceptualization, supervision.

## Funding

The authors have nothing to report.

## Ethics Statement

The authors have nothing to report.

## Conflicts of Interest

The authors declare no conflicts of interest.

## Data Availability

Data sharing is not applicable to this article as no datasets were generated or analyzed during the current study.
